# Evaluating a School-Based Public Health Intervention for Self-Management in Children with Atopic Dermatitis: A Non-Randomized Controlled Study

**DOI:** 10.3390/children12060676

**Published:** 2025-05-24

**Authors:** Jinhee Seo, Gaeun Kim

**Affiliations:** 1 Graduate School, Keimyung University, Dalgubeol-daero, Dalseo-gu, Daegu 1095, Republic of Korea; 2 College of Nursing, Keimyung University, Dalgubeol-daero, Dalseo-gu, Daegu 1095, Republic of Korea

**Keywords:** dermatitis, atopic, child, self-management

## Abstract

Purpose: This study evaluated the effectiveness of a school-based experiential self-management program for children with atopic dermatitis (AD) based on Roy’s adaptation theory. Design and Methods: Data were collected from June to August 2021, with 33 children in the experimental group and 32 in the control group. Participants were 10- to 11-year-old elementary school children who reported having AD symptoms within the past year and were able to complete self-report questionnaires. The program consisted of seven weekly school-based sessions that included disease education, symptom management techniques, skin care practices, nutritional guidance, and self-esteem enhancement activities. Outcomes, including AD severity, disease-related knowledge, adaptive behavior, self-esteem, and quality of life, were measured at baseline, post-intervention, and four weeks post-intervention using Generalized Estimating Equation analysis. Results: The experimental group showed significant improvements in AD severity (SCORAD: 22.80 ± 3.18 to 17.75 ± 2.24), disease-related knowledge (10.64 ± 2.00 to 13.64 ± 1.39), adaptive behavior (3.55 ± 1.70 to 10.58 ± 2.45), self-esteem (26.18 ± 4.76 to 31.55 ± 3.46), and quality of life (90.24 ± 11.07 to 100.27 ± 9.76), while the control group remained unchanged. Improvements were sustained four weeks post-intervention. Conclusions: This program effectively reduced AD severity and enhanced knowledge, adaptive behavior, self-esteem, and quality of life in children with AD. Practice Implications: School-based self-management programs effectively enhance disease knowledge, adaptive behaviors, and quality of life in children with AD.

## 1. Introduction

Atopic dermatitis (AD) is a common chronic inflammatory skin disease in children, marked by persistent itching, dry skin, and eczema due to allergic reactions [1,2]. AD affects about 10% of school-aged children in the U.S., 15.5% in Europe, and 10.3% in Japan [3]. Beyond physical symptoms, severe itching from AD disrupts learning, emotional well-being, and sleep, limiting children’s engagement in daily activities [4,5]. Frequent skin injuries can lead to negative self-image and psychosocial issues, resulting in a lower quality of life compared to children with other chronic conditions [6,7,8,9,10]. As AD symptoms fluctuate with allergen exposure, daily self-management is essential [11,12].

School-age children can perform self-management sufficiently because it is the time to learn and act so that they can recognize their health status through specific manipulations and active thinking in cognitive development and control their health behaviors by themselves [13,14]. Self-management education during this period significantly influences children’s health attitudes and behaviors, forming the foundation for sustainable health habits throughout life [13]. In school-age children, the influence of teachers and peers is important, and many daily activities are carried out in schools [13,14,15]. The recent international literature also supports the need for structured self-management in eczema and the appropriateness of school-based interventions. A Cochrane review highlighted that self-management interventions significantly improve outcomes in people with eczema [16]. Moreover, the WHO Health Promoting School framework has been shown to support student health and academic achievement through structured school-based programs [17]. Therefore, school-based programs are effective in enhancing student participation and performance skills [15,16,17]. Additionally, school-based health programs have positive aspects in children’s access to medical services, health equity, and cost benefits as teachers play two roles: treatment and education [18].

Prior studies for symptom management of children with AD were on the effects of alternative therapy [19,20], patient and caregiver education [21,22], and group education [23,24] in outpatient hospitals. However, studies suggest that school-based education programs combining knowledge and practical skills are more effective than hospital-based interventions, particularly in improving disease self-management for children with AD [23,25]. In addition, Staab et al. [23] proposed the development and application of a structured program suitable for each age group of children and adolescents with AD. Therefore, there is a need for research on the development of programs to improve the quality of life and practice and implementation of self-management for children with AD in their daily lives [23,26,27].

In Roy’s theory of adaptation, nursing is the one that promotes human adaptation and health to environmental stimuli [28]. This theoretical framework provides a basis for designing interventions that promote adaptive responses by enhancing individuals’ capacity to cope with internal and external stimuli. In this study, based on Roy’s adaptation theory, a school-based self-management program consisting of understanding and experiential activities on diseases considered as coping mechanisms is positively adapted to environmental stimuli (disease severity, disease-related knowledge and adaptive behavior, general characteristics) and attempted to confirm that the quality of life was improved.

This study aimed to evaluate the effectiveness of a school-based experiential activity-centered self-management program for school-aged children with AD, grounded in Roy’s adaptation theory. The objective was to provide empirical evidence regarding improvements in disease severity, disease-related knowledge, adaptive behavior, self-esteem, and quality of life.

## 2. Methods

### 2.1. Study Design, Registration, and Ethical Approvals

This study employed a quasi-experimental design with a non-equivalent control group and repeated measures at three timepoints (pre-test, post-test, and follow-up). Participants were assigned to either the experimental or control group by school, not by random allocation.

### 2.2. Setting

After this researcher met with the head of the Center for Atopy and Asthma Education Information Center in D city and the person in charge and explained the purpose of this study, 10 atopy and asthma safety schools were recommended. We visited 10 recommended schools and explained the purpose and content of this study to the principal and health teachers. As a result of requesting cooperation for the study, there were 5 schools that agreed to participate in the study, and 3 experimental groups and 2 control groups were assigned by random allocation.

### 2.3. Study Population

Participants of the study were children aged 10 to 11 who responded that they had AD in the past 12 months in the 2021 AD prevalence survey: (1) SCORAD index less than 50; (2) children who do not participate in AD management programs at other medical institutions; (3) children who have no problem filling out their own questionnaire; (4) consent to be available for the follow-up assessments. Children were excluded if they had cognitive impairments or communication difficulties that would prevent participation in educational activities or completion of self-report questionnaires. Those currently receiving other clinical treatments for AD outside of school settings were also excluded. The information and consent form for the study were sent by home correspondence. Consequently, both parents and children agreed to participate in the study with 38 experimental groups and 38 control groups.

### 2.4. Sample Size and Power Calculation

**Figure 1:**
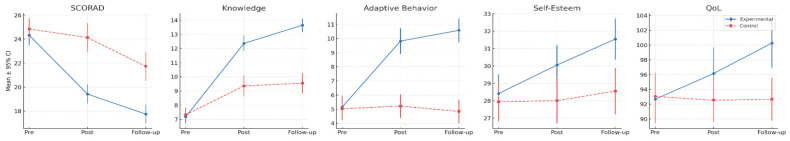
Outcomes at pre, post, and follow-up with 95% CI.

For the sample size of this study, the G-power 3.1.9.2 [29] program was used. The number of subjects was calculated based on the significance level (α) = 0.05, power of verification (1-β) = 0.80, and effect size (d) = 0.15. Thus, a total of 56 subjects were required for 28 subjects per group, 38 subjects for the experimental group and the control group, and a total of 76 subjects were selected in consideration of a 30% dropout rate. During the course of the study, 5 people from the experimental group and 6 people from the control group were eliminated, and a total of 65 people in the final experimental group and 32 controls participated in the study (Figure 1).

### 2.5. Intervention

Participants in this study were children with AD from five elementary schools in D city, with 33 in the experimental group and 32 in the control group. Data collection occurred between 8 June and 21 August 2021. The experimental group participated in a school-based experiential self-management program consisting of seven weekly 40-minute sessions from 8 June to 24 July, while the control group received no intervention.

The program was conducted over seven sessions, each with a specific focus designed to enhance self-management skills and quality of life for children with AD. The first session aimed to foster emotional connection and create a supportive environment among participants. In the second session, educational content was provided to help children understand the causes, symptoms, management, and prevention of AD. The third session focused on teaching techniques for managing itching behaviors, including the application of ointments, the use of wet dressings, and behavior modification strategies. The fourth session guided participants in identifying exacerbation triggers through practical activities, such as creating cotton masks. The fifth session emphasized proper skin care practices, including effective bathing methods and hands-on activities like preparing moisturizers. The sixth session addressed nutrition management, educating participants on beneficial and harmful foods and training them to monitor food additives. The final session aimed to promote self-esteem through activities such as drawing body images and engaging in storytelling to foster a positive self-concept. Each session was carefully structured to integrate cognitive and practical elements, ensuring that children could apply the skills learned in their daily lives. The program was delivered by a board-certified pediatric nurse who had over five years of clinical experience and had previously provided group education for children with AD in both hospital and community settings.

The educational approach was based on Roy’s adaptation theory, categorizing learning stages into cognitive and regulatory mechanisms [30,31]. The content and structure of the program were developed based on Roy’s adaptation theory and the previous literature on educational interventions for children with AD. Each session was designed to integrate cognitive and behavioral components in alignment with the theory’s focus on promoting adaptive responses to environmental stimuli. This approach aimed to enhance the children’s ability to manage chronic symptoms within their daily school environment. The cognitive mechanism included perception, learning, and emotional understanding, applied in each session’s introduction. The regulatory mechanism involved physical responses through experiential activities, forming the development stage, followed by the evaluation of learning outcomes.

Each session, apart from the initial introduction and “Understanding AD”, followed a structure of introduction (10 min lecture), development (25 min activity), and finishing (5 min evaluation). Classes took place in familiar classroom environments, emphasizing peer interactions (Table 1). To assess program effectiveness, AD severity, disease-related knowledge, adaptive behavior, self-esteem, and quality of life were measured at baseline, immediately post-intervention, and four weeks after the program concluded.

### 2.6. Outcome Measure

#### 2.6.1. Atopic Dermatitis Severity

The SCORing AD (SCORAD) index was determined based on the range of lesions (A), the intensity of lesions (B), and the subjective symptoms (C). A was calculated for the inflammatory lesion by applying the “rule of nine”, and the maximum score is 100. B was calculated by evaluating six symptoms (such as erythema, abrasion, edema/papule, exudate/crust, dryness, and lichenification) as 0–3 points, constituting a maximum score of 18. C was calculated by scoring the level of pruritus and sleep disorder as 0–10 points, making a maximum score of 20. The generic SCORAD was calculated by a formula of “A/5 + 7B/2 + C”, and its maximum score is 103. AD was classified, according to the SCORAD index score, into mild: SCORAD < 25, moderate: 25–50, and severe: SCORAD > 50 [25].

#### 2.6.2. Disease-Related Knowledge

The disease-related knowledge was measured using a questionnaire developed by researchers based on the “2016 Counseling Manual on Atopy & Asthma” [31] and a previous study [32] and verified by an expert panel. A total of 15 Yes/No items for understanding AD (n = 2), symptom (n = 2), nutrition (n = 2), shower (n = 3), moisturizing (n = 2), skin care (n = 2), clothing (n = 1), and medicine (n = 1) were responded, and 1 and 0 points were assigned to correct and incorrect answers, respectively, constituting a maximum score of 15. The higher scores indicate higher levels of disease-related knowledge, and the reliability was Cronbach’s α = 0.71.

#### 2.6.3. Adaptive Behavior

Adaptive behavior was measured using a 14-item questionnaire developed from the 2016 Counseling Manual on Atopy & Asthma and previous studies [31,32], validated by an expert panel. Items included shower frequency, duration, method, water temperature, soap type, towel-drying method, moisturizer use, sun protection, nail length, clothing choices, exercise habits, post-exercise hygiene, and nutrition. Each item matched to adaptive behavior scored 1 point, with a maximum score of 14. Higher scores indicated greater adaptive behavior, with reliability of Cronbach’s α = 0.89.

#### 2.6.4. Self-Esteem

The self-esteem was measured using a scale developed by Rosenberg [33]. This scale consisted of ten items, of which six were positive (items 1, 2, 4, 6, and 8), and five were negative (items 3, 5, 8, 9, and 10), and responded using the four-point Likert scale from 1 (rarely) to 4 (always). The negative ones were reversely coded, resulting in a total score ranging from 10 to 40. The higher scores indicate higher levels of self-esteem, and the reliability was Cronbach’s α = 0.82

#### 2.6.5. Quality of Life

The quality of life of pediatric patients with AD was measured using the PedsQLTM 4.0 Generic Core Scale developed by Varni et al. [34]. This scale comprises 23 items divided into physical functioning (n = 8), emotional functioning (n = 5), social functioning (n = 5), and school functioning (n = 5), and they responded using the five-point Likert scale from 1 (never) to 5 (always). The generic points ranged from 23 to 115, and higher scores indicate a higher quality of life. The reliability was Cronbach’s α = 0.82 for the original version and 0.93 in this study.

#### 2.6.6. Statistical Analysis

Data analysis was conducted using SAS 9.4. Demographic, and dependent variables were analyzed for frequency, percentage, mean, and standard deviation. To confirm homogeneity between the experimental and control groups, χ^2^ and Fisher’s exact tests were used. The Kolmogorov–Smirnov test checked the normality of dependent variables, followed by independent *t*-tests for normally distributed data, while non-normally distributed variables were analyzed with the Mann–Whitney U test. To evaluate differences before and after the intervention, the generalized estimating equation (GEE) was applied using SAS PROC GENMOD.

## 3. Results

### 3.1. Participant Characteristics

Participant demographics are summarized in Table 2, showing no significant differences between groups after randomization. Family history of AD was most common among siblings (48.5% experimental, 40.6% control). Asthma affected 3.0% of the experimental and 9.4% of the control group, while allergic rhinitis was reported by 66.7% and 78.1%, respectively. Pet ownership was similar between groups (36.4% experimental, 31.3% control), and breastfeeding was reported by 75.8% of the experimental group and 62.5% of the control group. No significant differences were found between the experimental and control groups in baseline characteristics, confirming group homogeneity (Table 2).

### 3.2. Atopic Dermatitis Severity

AD severity in the experimental group decreased significantly from 22.80 ± 3.18 (pre-test) to 18.34 ± 2.53 (post-test) and 17.75 ± 2.24 (follow-up), while the control group showed no change (21.85 ± 3.49 to 21.73 ± 3.52). Statistically significant differences were observed between groups (Wald χ^2^ = 3.11, *p* = 0.002) and over time in both post-test (Wald χ^2^ = −2.66, *p* = 0.008) and follow-up (Wald χ^2^ = −3.44, *p* = 0.001). Interaction effects indicated continued improvement in the experimental group over time (Wald χ^2^ = −10.35, *p* < 0.001; Wald χ^2^ = −8.69, *p* < 0.001) (Table 3).

### 3.3. Disease-Related Knowledge

The disease-related knowledge of the experimental group increased to 10.64 ± 2.00 in the pre-test, 13.64 ± 1.27 in the post-test, and 13.64 ± 1.39 in the follow-up test. The control group was 9.88 ± 2.23 in the pre-test, 9.91 ± 2.02 in the post-test, and 9.5 ± 2.10 in the follow-up test. There was no significant difference between groups, there was no significant difference in the post-test over time, and there was a statistically significant difference in the follow-up test (Wald χ^2^ = −2.32, *p* = 0.020). In the interaction between the group and time, the knowledge related to disease of the experimental group was significantly increased by the post-test (Wald χ^2^ = 17.10, *p* < 0.001) and the follow-up test (Wald χ^2^ = 8.05, *p* < 0.001) over time (Table 3).

### 3.4. Adaptive Behavior

The adaptive behavior of the experimental group increased to 3.55 ± 1.70 in the pre-test, 10.27 ± 2.05 in the post-test, and 10.58 ± 2.45 in the follow-up test. The control group was 4.72 ± 2.58 for the pre-test and 4.66 ± 2.47 for the post-test. In the follow-up test, there was no significant change, with 4.84 ± 2.42 points. There was no significant difference between groups, and there was no significant difference over time between the post-test and the follow-up test.

In the interaction between the group and time, the adaptive behavior of the experimental group was statistically significantly higher in the post-test (Wald χ^2^ = 13.68, *p* < 0.001) and follow-up test (Wald χ^2^ = 10.58, *p* < 0.001) over time (Table 3).

### 3.5. Self-Esteem

The self-esteem of the experimental group increased to 26.18 ± 4.76 in the pre-test, 31.61 ± 4.32 in the post-test, 31.55 ± 3.46 in the follow-up test, 30.25 ± 4.64 in the pre-test, and 29.19 ± 4.52 in the post-test in the control group. In the follow-up test, there was a tendency to decrease to 28.56 ± 3.87. There was no significant difference between groups, and there was no significant difference over time in the post-test and follow-up test. In the interaction between the group and time, the self-esteem of the experimental group was statistically significantly higher in the post-test (Wald χ^2^ = 5.10, *p* < 0.001) and follow-up test (Wald χ^2^ = 2.69, *p* = 0.007) over time (Table 3).

### 3.6. Quality of Life

Quality of life in the experimental group improved from 90.24 ± 11.07 (pre-test) to 101.73 ± 10.21 (post-test) and remained high at 100.27 ± 9.76 (follow-up). The control group showed little change, from 94.91 ± 9.88 to 93.38 ± 9.09, with a slight decrease to 92.66 ± 8.32 at follow-up. While no significant differences were observed between groups overall, the experimental group showed significantly higher quality of life in both post-test (Wald χ^2^ = 12.11, *p* < 0.001) and follow-up (Wald χ^2^ = 7.25, *p* < 0.001) (Figure 1, Table 3).

## 4. Discussion

This study confirmed the effect of a school-based experiential activity-centered self-management program on severity, knowledge, adaptive behavior, self-esteem, and quality of life in school-aged children with AD. Previous studies applying programs on AD were mainly limited to alternative therapies [19,20] or education [21,22,23]. However, self-management is important for atopy, and the school-age period is appropriate for education on self-management [13]. Furthermore, school-based education is more effective [23,25,26,27], and learning from experiences with peers is also effective [13,15]. Therefore, this study made a significant contribution by applying a school-based experiential activity-centered self-management program to school-aged children and evaluating its effects.

In this study, the school-based experiential activity-centered self-management program significantly reduced the atopic severity score in school-aged children with AD, as has also been reported in previous studies [26,27,35]. Based on Roy’s adaptation model, an explanation for this result is that experiential activities made the participants recognize and adapt to their symptoms, prompting them to tackle and manage them well by themselves. However, the severity score in this study may have been different from those reported in previous studies because this study included children with mild symptoms who answered that they had symptoms in an AD prevalence survey conducted at school, whereas previous studies included children diagnosed with AD in a medical setting. Nevertheless, for the management of AD, which involves repeated cycles of exacerbation and remission, experiential education-oriented management with peers in the school environment, where children spend most of their time, may be effective.

Although the reduction in SCORAD scores was statistically significant in the experimental group, the mean difference (5.05 points) did not reach the minimal clinically important difference (MCID) of 8.7 points, as defined by Schram et al. [36]. Therefore, the clinical relevance of this improvement should be interpreted with caution. Furthermore, no established MCID has been validated for pediatric self-esteem using the Rosenberg scale, limiting the interpretation of observed improvements in this variable.

Furthermore, this program significantly improved disease-related knowledge in children with AD, which is consistent with previous studies [32,37]. This result suggests that school-aged children are in a period when independent health management is possible and that providing knowledge about causes, symptoms, treatment, and management methods at this age can help children engage in self-management.

Moreover, this program was significantly effective in enhancing the adaptive behaviors of children with AD. This result supports the findings of Son et al. [32] that lifestyle habits exhibited statistically significant positive changes both immediately after and 3 months following a camp for elementary school students with AD, as well as previous studies reporting that education combining various experiential activities remarkably reduced the frequency of exacerbation of the disease [32,37,38]. An explanation for this result may be that the self-management program, which consisted of experiential activities that can be practiced in one’s daily life as correct behaviors for AD itching, led to significant positive adaptive behavioral changes. In particular, since acquired risk factors related to health status and lifestyle habits can negatively influence AD [39], it is necessary to suggest proper intervention strategies that address those risk factors.

This study found a significant improvement in self-esteem among children with AD, aligning with previous research [5,6,23,27,40,41]. The “drawing my body image” activity helped enhance self-esteem by fostering unity, self-confidence, and positive body images through group storytelling. Since AD often impacts body image and self-esteem due to itching and skin damage [5,6,41], educational interventions like this are essential. The program also improved quality of life, consistent with studies [23,27] showing better outcomes in groups receiving structured, practice-based education compared to information-only interventions. This finding contrasts with studies where one-time consultations showed no effect, likely due to the program’s experiential, activity-centered approach.

This study suggests implementing structured, experiential self-management programs for children with AD in schools. However, as effects were measured only up to 4 weeks post-program, long-term impacts remain uncertain, and further research is needed. Since participants were from specific regions, results may not generalize to all elementary students, so replication studies across varied regions are recommended.

Although the findings of this study are meaningful, several limitations should be noted. First, the follow-up period was limited to four weeks after the intervention, which may not fully capture the long-term sustainability of the observed effects. Second, the participants were recruited from a limited geographical region, which may restrict the generalizability of the results to broader populations. Third, due to the nature of the intervention and the school setting, blinding was not possible, which could have introduced bias in reporting or behavior. Future studies with longer follow-up durations, more diverse populations, and randomized controlled designs are recommended to validate and expand upon these findings.

## 5. Conclusions

The school-based experience-based self-management program in this study was verified as an effective intervention for reducing the severity of AD and improving disease-related knowledge, adaptive behavior, self-esteem, and quality of life in school-aged children with AD. In the future, by applying the school-based experience-based self-management program of this study to the school field, it will become a guideline for health teachers to improve the self-management ability of children with AD.

## Figures and Tables

**Table 1:** School-based experiential activity-centered self-management program.

Session	Coping Mechanism	Time	Main Content	Method	Contents of Each Session	Relation with the Conceptual Framework
1	Cognator	40	Pleasant meeting	Lecture	Introduction of program purpose and contents, pre-survey Check of physical condition and management method	Focal stimulus (AD severity) Situational stimulus (disease-related knowledge, adaptive behavior)
2	Cognator	40	Understanding of AD	Lecture	Introduction of AD causes, symptoms, and management and prevention methods	Role functioning style (disease-related knowledge, adaptive behavior
3	Cognator	10	Behavior modification methods regarding itch coping	Lecture	Explanation of behavior modification methods for removing the vicious cycle; inflammation→itch→scratching→inflammation→itch	Focal stimulus (AD severity) Situational stimulus (disease-related knowledge, adaptive behavior)
	Regulator	25	Behavior modification methods	Activity	Experience of spreading a gauze soaked in normal saline solution, spreading moisturizing cream, and spreading ointment	Role functioning style (disease-related knowledge, adaptive behavior)
	Cognator	5	FA, NS	Lecture	Guidance of exacerbation factor and trigger	
4	Cognator	10	Exacerbation factor and trigger of AD	Lecture	Explanation of exacerbation factor and trigger	Situational stimulus (disease-related knowledge, adaptive behavior)
	Regulator	25	Making an atopy care product	Activity	Instructing students to wear clothes made from cotton after an experiment comparing moisture absorption between cotton and nylon Making a cotton sanitary pad or an atopy care product	Role functioning style (disease-related knowledge, adaptive behavior)
	Cognator	5	FA, NS	Lecture	Guidance of skin care methods	
5	Cognator	10	Skin care methods for AD patients	Lecture	Explanation of how to take a bath and method Inducing students to form the “133” habit in ordinary life	Role functioning style (adaptive behavior)
	Regulator	25	133 method How to wipe moisture	Activity	Practice of the 133 method and how to wipe moisture Experience of making a moisturizer	Role functioning style (disease-related knowledge, adaptive behavior)
	Cognator	5	FA, NS	Lecture	Guidance of foods that are good and bad for AD	
6	Cognator	10	Nutrition management	Lecture	Explanation of foods that are good and bad for AD	Situational stimulus (disease-related knowledge)
	Regulator	25	Experience of removing artificial pigments	Activity	Experience of removing artificial pigments and additives Investigation of food additives and presentation of things that we have to do	Role functioning style (adaptive behavior)
	Cognator	5	FA, NS	Lecture	Understanding of my body image	
7	Cognator	10	Self-esteem Understanding my body image	Lecture	Checking the parts that hurt and are healthy in body and mind	Focal stimulus (AD severity)
					Encouraging students to recognize themselves properly	Self-concept
	Regulator	25	My body image expression activity	Activity	Storytelling of body image and what to express on the drawing paper	Role functioning style (disease-related knowledge, adaptive behavior)
					Encouraging students to recognize a right self-image and form self-esteem	Self-concept
	Cognator	5	FA	Lecture	Assessment of feelings Guidance of survey	

AD = atopic dermatitis; FA = formative assessment; NS = notice of session.

**Table 2:** Demographic and baseline characteristics of participants.

Characteristic	Categories	Exp (n = 33)	Con (n = 32)	χ^2^	*p*
		n (%)	n (%)		
Gender	Male	16(48.5)	12(37.5)	0.80	0.371 *
	Female	17(51.5)	20(62.5)		
Age	11	19(57.6)	17(53.1)	0.13	0.718 *
	10	14(42.4	15(46.9)		
Marriage status of parents	Married	28(84.8)	23(71.9)	1.62	0.203 *
	Divorced or bereaved	5(15.2)	9(28.1)		
Subjective economic status	High	7(21.2)	5(15.6)	1.29	0.618 ^†^
	Medium	25(75.8)	24(75.0)		
	Low	1(3.0)	3(9.4)		
Type of residence	Apartment	18(54.5)	25(78.1)	3.47	0.062 *
	House	15(45.5)	7(21.9)		
Academic record	High	13(39.4)	13(40.6)	0.83	0.660 *
	Medium	12(36.4)	14(43.8)		
	Low	8(24.2)	5(15.6)		
Family history ^‡^	Father	6(18.2)	3(9.4)	1.06	0.475 ^†^
	Mother	10(30.3)	5(15.6)	1.97	0.160 *
	Siblings	16(48.5)	13(40.6)	0.41	0.524 *
Allergic disease ^‡^	Asthma	1 (3.0)	3 (9.4)	1.13	0.355 ^†^
	Allergic rhinitis	22(66.7)	25(78.1)	1.07	0.302 *
Pet	Pet	12(36.4)	10(31.3)	0.19	0.663 *
	No pet	21(63.6)	22(68.7)		
Breast-feeding	Breast-feeding	25(75.8)	20(62.5)	1.34	0.247 *
	No breast-feeding	8(24.2)	12(37.5)		

* Chi-square test; ^†^ Fisher’s exact test; ^‡^ duplicate answers; Exp = experimental group; Con = control group.

**Table 3:** Effects of the school-based experiential activity-centered self-management program.

Variable	Participant	Pre	Post	Follow-Up	Source	Esti-mate	SE	Wald χ^2^	*p*
		M ± SD	M ± SD	M ± SD					
Atopic Dermatitis Severity	Exp (n = 33) Con (n = 32)	22.80 ± 3.18 21.85 ± 3.49	18.34 ± 2.53 21.85 ± 3.70	17.75 ± 2.24 21.73 ± 3.52	G	4.47	1.44	3.11	0.002
					T(P)	−0.65	0.24	−2.66	0.008
					T(F)	−0.81	0.24	−3.34	0.001
					G*(P)	−4.86	0.47	−10.35	<0.001
					G*T(F)	−6.19	0.71	−8.69	<0.001
Disease-related Knowledge	Exp (n = 33) Con (n = 32)	10.64 ± 2.00 9.88 ± 2.23	13.64 ± 1.27 9.91 ± 2.02	13.64 ± 1.39 9.55 ± 2.10	G	−0.71	0.40	−1.76	0.078
					T(P)	0.12	0.12	1.05	0.293
					T(F)	−0.34	0.15	−2.32	0.020
					G*(P)	4.79	0.28	17.10	<0.001
					G*T(F)	3.34	0.42	8.05	<0.001
Adaptive Behavior	Exp (n = 33) Con (n = 32)	3.55 ± 1.70 4.72 ± 2.58	10.27 ± 2.05 4.66 ± 2.47	10.58 ± 2.45 4.84 ± 2.42	G	−0.95	0.53	−1.80	0.072
					T(P)	0.34	0.29	1.16	0.247
					T(F)	0.54	0.30	1.81	0.071
					G*(P)	6.49	0.48	13.68	<0.001
					G*T(F)	6.45	0.61	10.58	<0.001
Self-Esteem	Exp (n = 33) Con (n = 32)	26.18 ± 4.76 30.25 ± 4.64	31.61 ± 4.32 29.19± 4.52	31.55 ± 3.46 28.56 ± 3.87	G	−3.06	1.92	−1.59	0.112
					T(P)	−0.32	1.17	−0.28	0.782
					T(F)	−0.93	1.61	−0.81	0.420
					G*(P)	5.83	1.14	5.10	<0.001
					G*T(F)	5.56	2.07	2.69	0.007
Quality of Life	Exp (n = 33) Con (n = 32)	90.24 ± 11.07 94.91 ± 9.88	101.73 ± 10.21 93.38 ± 9.09	100.27 ±9.76 92.66 ± 8.32	G	−3.15	2.87	−1.10	0.272
					T(P)	−0.63	1.04	−0.60	0.547
					T(F)	−1.23	1.17	−1.05	0.292
					G*(P)	12.28	1.01	12.11	<0.001
					G*T(F)	11.04	1.52	7.25	<0.001

G = group; T(P) = time (post); T(F) = time (follow-up); Exp = experimental group; Con = control group, the asterisk (*) indicates the interaction effect between group and time.

## Data Availability

The data presented in this study are available on request from the corresponding author. The data are not publicly available due to privacy issues.
